# Advanced Maternal Age Affects the Cryosusceptibility of Ovulated but not In Vitro Matured Mouse Oocytes

**DOI:** 10.1007/s43032-024-01462-6

**Published:** 2024-01-31

**Authors:** Akshatha Daddangadi, Shubhashree Uppangala, Shama Prasada Kabekkodu, Nadeem Khan G, Guruprasad Kalthur, Riccardo Talevi, Satish Kumar Adiga

**Affiliations:** 1 Centre of Excellence in Clinical Embryology, Department of Reproductive Science, Kasturba Medical College, Manipal Academy of Higher Education, Manipal, 576 104 India; 2 Division of Reproductive Genetics, Department of Reproductive Science, Kasturba Medical College, Manipal Academy of Higher Education, Manipal, 576 104 India; 3 Department of Cell and Molecular Biology, Manipal School of Life Sciences, Manipal Academy of Higher Education, Manipal, 576 104 India; 4 Division of Reproductive Biology, Department of Reproductive Science, Kasturba Medical College, Manipal Academy of Higher Education, Manipal, 576 104 India; 5 Dipartimento Di Biologia, Università Di Napoli “Federico II”, Complesso Universitario Di Monte S Angelo, Naples, Italy

**Keywords:** Assisted reproductive technology, Fertility preservation, In vitro maturation, Maternal age, Oocyte vitrification

## Abstract

Oocyte cryopreservation is offered to women of various age groups for both health and social reasons. Oocytes derived from either controlled ovarian stimulation or in vitro maturation (IVM) are cryopreserved via vitrification. As maternal age is a significant determinant of oocyte quality, there is limited data on the age-related susceptibility of oocytes to the vitrification-warming procedure alone or in conjunction with IVM. In the present study, metaphase II oocytes obtained from 2, 6, 9, and 12 month old *Swiss albino* mice either by superovulation or IVM were used. To understand the association between maternal age and oocyte cryotolerance, oocytes were subjected to vitrification-warming and compared to non vitrified sibling oocytes. Survived oocytes were evaluated for mitochondrial potential, spindle integrity, relative expression of spindle checkpoint protein transcripts, and DNA double-strand breaks. Maturation potential and vitrification-warming survival were significantly affected (*p* < 0.001 and *p* < 0.05, respectively) in ovulated oocytes from the advanced age group but not in IVM oocytes. Although vitrification-warming significantly increased spindle abnormalities in ovulated oocytes from advanced maternal age (*p* < 0.01), no significant changes were observed in IVM oocytes. Furthermore, *Bub1* and *Mad2* transcript levels were significantly higher in vitrified-warmed IVM oocytes (*p* < 0.05). In conclusion, advanced maternal age can have a negative impact on the cryosusceptibility of ovulated oocytes but not IVM oocytes in mice.

**Supplementary Information:**

The online version contains supplementary material available at 10.1007/s43032-024-01462-6.

## Introduction

Oocyte cryopreservation is offered to women of various age groups for both health and social reasons. Oocytes derived from either controlled ovarian stimulation or in vitro maturation are cryopreserved. Although vitrification is considered the preferred freezing method, reports have suggested its detrimental effects on oocyte viability, meiotic spindle, chromatin, and cytoskeletal integrity [1]. Maternal age significantly affects oocyte quality by inducing spindle assembly checkpoint dysregulation, cohesion deterioration, and mitochondrial dysfunction, leading to oocyte aneuploidy [2–4]. A recent study has demonstrated that maternal age influences fertilisation, cell polarity, and blastocyst formation [5]. The quality was significantly impaired when such oocytes were subjected to vitrification-warming [6].

IVM of oocytes is a laboratory technique wherein germinal vesicle (GV) oocytes retrieved from the ovary with minimal or no gonadotropin priming resume meiosis, progress to metaphase II (MII) oocytes, and can fertilise. IVM of oocytes has several benefits like reduced cost, minimal gonadotropin priming, and a simplified treatment procedure [7].Clinically, IVM is offered to patients at risk of developing ovarian hyperstimulation syndrome (OHSS), such as women with polycystic ovarian syndrome (PCOS), women in need of immediate fertility preservation due to oncofertility indications, and prepubertal girls who cannot undergo ovarian stimulation due to the inactive pituitary hypothalamic axis [8, 9]. IVM of GV and metaphase I oocytes retrieved during the conventional controlled ovarian hyperstimulation (COH) cycle have established good pregnancy outcomes [10]. Several studies comparing the efficiency of ovulated and IVM oocytes elucidated that in vivo and IVM oocytes were predominantly but not entirely similar [11]. Though IVM oocytes exhibit a higher percentage of spindle and chromosomal abnormalities than ovulated oocytes in humans [12], the cryotolerance of ovulated and IVM oocytes is proportional [13].

Advanced maternal age is considered a risk factor for poor ovarian response. As oocytes retrieved from young and old mice showed no association with age or IVM potential [14], it is clinically beneficial if IVM of oocytes can be a preferred alternative for ovarian stimulation in advanced maternal age groups. IVM and oocyte cryopreservation are intimately related to standard assisted reproductive technology (ART) practices. This study aimed to investigate age-dependent changes in the structural and functional integrity of IVM oocytes as well as their capacity to tolerate vitrification-warming induced stress. We hypothesised that the additional intervention of IVM can make oocytes from advanced maternal age more prone to vitrification-warming induced changes than non-IVM derived oocytes.

Earlier studies have demonstrated that maternal age and vitrification can influence spindle morphology and checkpoint regulators, such as *Bub1 and Mad2* [15, 16]. Oocyte spindle abnormalities can subsequently result in perturbations in mitochondrial function [17] and DNA damage [18]. Therefore, using mice as an experimental model, this study compared the cryosusceptibility of ovulated and IVM oocytes with maternal age by evaluating spindle integrity using immunofluorescence and qPCR, mitochondrial potential using the cationic carbocyanine fluorescence dye JC-1, and DNA damage using γ-H2AX immunofluorescence, a molecular marker of DNA damage.

## Materials and Methods

All chemicals were purchased from Sigma-Aldrich ( USA), unless otherwise stated.

### Animals and Oocyte Collection

The institutional animal ethics committee of Kasturba Medical College, Manipal, approved the experimental procedures (IAEC/KMC/46/2019). Healthy female *Swiss albino* mice of 2,6,9 and 12 months of age, representing 20, 30, 36 and 42.5 human years [19] were maintained at 25 ± 2 °C temperature, 45–55% humidity, 12:12 h photoperiod, and unrestricted access to standard diet and water ad libitum were used for the experiments. Animals were sacrificed by cervical dislocation. Ovaries were excised from the posterior region of the abdominal cavity using micro-scissors. GV oocytes for the in vitro maturation were obtained by gently teasing the cortical region of the ovaries using fine needles in prewarmed M2 medium under a microscope at 37 °C. The oocyte cumulus complex (OCC) was retrieved successfully from different study groups primed with 5 IU PMSG (Pregnant mare serum gonadotropin PMSG; Cat. No# HOR-272, ProSpec-Tany TechnoGene Ltd., Israel) and 10 IU hCG (Human chorionic gonadotropin, Cat. No# Lupi-HCG 2000, Lupin, India), injected after 48 h of PMSG administration. The primed mice were sacrificed 12-14 h post hCG injection to collect OCC in EBSS medium (Cat. No# E2888) from the ampulla and denuded enzymatically by treatment with 1 mg/ml hyaluronidase (Cat. No# H4272) for 30 s and transferred to M16 medium (Cat. No# M7292). Metaphase II oocytes with intact plasma membranes and zona pellucida were considered for further experiments.

### In Vitro Maturation (IVM) of Oocytes

Cumulus free GV oocytes released from the ovaries were cultured in 20µL of DMEM media supplemented with 1% insulin transferrin selenium, 1% non-essential α-amino acids, 0.05% pyruvate, 0.05% penicillin–streptomycin, and 0.3% bovine serum albumin (BSA), as described in our previous studies [20, 21]. Oil overlaid medium droplets were cultured in a humidified atmosphere at 37 °C and 5%CO_2_ in a Heracell 150i incubator for 24 h. The maturation potential of the oocytes was measured by their ability to progress to metaphase Ι (oocytes with GV breakdown and without polar body) and metaphase ΙΙ (oocytes with one polar body were extruded) under an inverted phase contrast microscope (40X, IX 73, Olympus, Japan).

### Vitrification and Warming

Ovulated sibling and IVM oocytes were divided into ovulated non vitrified (O-NV), ovulated vitrified (O-V), IVM non vitrified (I-NV), and IVM vitrified (I-V) groups. The numerical digits with these study groups denote the age of the animals. Oocyte vitrification was performed according to the standard Kitazato Cryotop® method. Briefly, oocytes were rinsed in basal solution (BS) for one minute, transferred to an equilibration solution (ES), and incubated for 12–15 min. Oocytes were then exposed to vitrification solution (VS) for 30 s and loaded onto a cryolock (8–10 oocytes/Cryolock), then plunged into liquid nitrogen and kept for 30 min. The oocytes were then warmed by immersing the cryolock in a prewarmed thawing solution (TS) and subsequently moved to a dilution solution (DS) and washing solution (WS) for 3 and 6 min, respectively. A survival check was performed by culturing oocytes in M16 medium (Cat. No# M1285) for 3 h. Only oocytes with a homogenous cytoplasm, intact plasma membrane, and zona pellucida were considered to survive and were used for further analysis.

### Mitochondrial Membrane Potential Measurement

The mitochondrial membrane potential of the oocytes was measured using the cationic carbocyanine fluorescence dye JC-1, as described previously [21]. Oocytes were incubated in M16 medium with 1 μg/mL JC-1 dye (5,5′,6,6′-Tetrachloro-1,1′,3,3′-tetraethyl-imidacarbocyanine iodide, Cat. No# T3168, Molecular Probes, Life Technologies, USA) for 30 min at 37 °C and 5%CO_2_, avoiding direct light. After 30 min, the excess stain was washed with M16 medium supplemented with 0.1% BSA and mounted on a glass slide. Oocytes were analysed immediately at 488 nm using a fluorescence microscope (Imager-A1, Zeiss, Gottingen, Germany). The images were captured under 40X using Q-Capture software (Media Cybernetics Inc., USA). Oocytes with higher mitochondrial potential fluoresce orange owing to the multimerisation of JC‐1 and the formation of "J‐aggregates. Simultaneously, oocytes with a decreased mitochondrial potential fluoresced green. Mitochondrial membrane potential was represented as an orange to green fluorescence ratio determined by ImageJ software using RGB Plugin (National Institute of Health, Bethesda, Maryland, USA).

### Meiotic Spindle Organisation Assessment

The integrity of the meiotic spindle, post vitrification-warming was studied as previously described with minor modifications [20]. Survived metaphase II oocytes were permeabilised in extraction buffer (50 mM KCL, 5 mM ethylenediaminetetraacetic acid disodium salt, 0.5 mM Magnesium chloride, 25 mM HEPES, 25% Glycerol, 2% Triton X-100, 20 μM phenyl methane sulphonyl fluoride, pH 6.75) at 37 °C for 40 min after multiple washes in PBS with 0.1% BSA to remove culture media. Permeabilised oocytes were fixed using ice-cold methanol for 15 min at -20 °C and blocked with 5% knockout serum and 0.25% Triton-X in PBS for 1 h at 37 °C to prevent non-specific antibody binding. Oocytes were incubated with a 1:100 diluted primary anti-αtubulin antibody (Cat. No# T9026), overnight at 4 °C. After three washes with the blocking solution, the oocytes were transferred to a 1:200 FITC-tagged goat anti-mouse IgG antibody (Cat. No# NB7535, Novus Biologicals, USA) for 2 h at room temperature. The chromosomes were stained in the final incubation step for 10 min with 4 µg/mL DAPI (4',6-Diamidino-2-phenylindole, Cat. No# D9542). Finally, oocytes were mounted on a clean glass slide and observed under the 100 × objective of the Leica-SP8-DMi8 confocal laser scanning microscope, and images were captured using Leica Application Suit X software. Spindle structures were classified as normal or abnormal based on tubulin organisation and chromosomal alignment [22, 23].

### Parthenogenetic Activation and γ-H2AX Immunostaining

The oocytes were parthenogenetically activated by incubating in pre-equilibrated Ca^2+^ and Mg^2+^ free M16 medium containing 10 mM strontium chloride for 15 min at 37 °C in 5% CO_2_ [24]. Later, oocytes were transferred to prewarmed 0.1% BSA supplemented M16 medium and cultured for 9 h at 37 °C in 5% CO_2._ The presence of one pronucleus (PN) and two extruded polar bodies (2 PB) under an inverted phase contrast microscope (40X, IX 73, Olympus, Japan) was regarded as a parthenogenetically activated oocyte. Activated oocytes were immunostained to detect phosphorylated **γ-**H2AX [25]. Acid Tyrode treated (30 s at 37 °C), zona free oocytes were fixed using 4% paraformaldehyde at room temperature for 30 min followed by permeabilisation using 0.1% (v/v) Triton X-100,1 h at 37 °C. Oocytes were incubated with a 1:20 anti-phospho-histone H2AX antibody (Cat. No# 05–636-1) overnight at 4 °C and transferred to FITC conjugated 1:25 diluted goat anti-mouse IgG (Cat. No# NB7535, Novus Biologicals, USA), 1 h at 37 °C in the dark. The DNA was counterstained with 4 μg/mL DAPI, mounted on a clean glass slide using an anti-fading medium, and observed under a 63X objective of a Leica-SP0DMi8 confocal laser scanning microscope. The number of γ-H2AX foci in each oocyte was manually counted and categorised as either small (< 1 µm diameter) or large (> 1 µm diameter). Images were acquired using Leica Application Suit X software.

### Relative Expression of *Bub1* and *Mad2* by Quantitative Real-Time PCR (qPCR)

Total RNA was isolated from at least 50 metaphase II oocytes in each study group using an RNAqueous Micro Kit (Cat No# AM1931, Ambion, Life Technologies, USA). Briefly, oocytes were disrupted by vigorous vortexing in lysis solution and incubated at 42 °C for 30 min, after which the lysate was mixed with 50µL 100% ethanol and passed through the microfilter cartridge via centrifugation for 10 s at maximum speed. RNA was then bound to the microfilter cartridge. The filter was washed using 180 µL of wash solution 1 and wash solution 2/3 for 10 s at the maximum speed. Finally, RNA was eluted into the microelution tube using 10ul elution solution, preheated to 95 °C, incubated for 5 min at room temperature, and eluted into the microfilter by centrifuging for 30 s at 12000 rpm. RNA was quantified using a NanoDrop spectrophotometre (Thermo Fisher Scientific, USA). The RNA concentration was normalised to the lowest concentration among the experimental groups, and first-strand cDNA was synthesised using a High Capacity cDNA Reverse Transcription (RT) Kit (Cat No# 4,374,966, Applied Biosystems, USA). 20µL reaction mixture consisted of RT buffer (2µL), dNTP (0.8µL), RT random primers (2µL), reverse transcriptase (1.0µL), RNase inhibitor (1.0µL), nuclease free water (3.2µL) and 10µL RNA sample and loaded to preprogrammed thermal cycler (Thermo Fisher Scientific, USA). cDNA templates were amplified by quantitative polymerase chain reaction (qPCR) using SYBR Green chemistry (Cat No# RR420A, DSS Takara Bio, India) in a StepOne™ Real-Time PCR System (Thermo Fisher Scientific, USA) where 20µL reaction mixture consisted of cDNA (1µL), forward and backward primer (0.4µL), SYBR Green master mix (10µL), ROX dye (0.4µL) and nuclease free water (7.8µL). The relative expression of spindle checkpoint proteins transcripts such as *Bub1*(budding uninhibited by benzimidazole) and *Mad2* (mitotic arrest deficient 2) was estimated using predesigned primers and normalised to *Actb* (Actin beta) and *Gapdh* (Glyceraldehyde phosphate dehydrogenase) housekeeping genes according to 2^−ΔΔCT^ methodology. The primer details are provided in Supplementary Table 1.

### Statistical Analysis

The variables are expressed as mean ± Standard Error of Mean (SEM). Statistical comparisons were made using one-way analysis of variance (ANOVA) (for normally distributed variables) and the Kruskal–Wallis test. Data were evaluated using the GraphPad InStat software (GraphPad Inc., La Jolla, CA, USA). The graphical representation of the data was prepared using Microcal Origin 8.0 software (Origin Lab Corporation, Northampton, MA, USA). Statistical significance was set at *p* < 0.05. Each experiment was repeated at least three times.

## Results

### Functional Competence and Cryosurvival of Ovulated Oocytes are Age Related

**Fig. 1:**
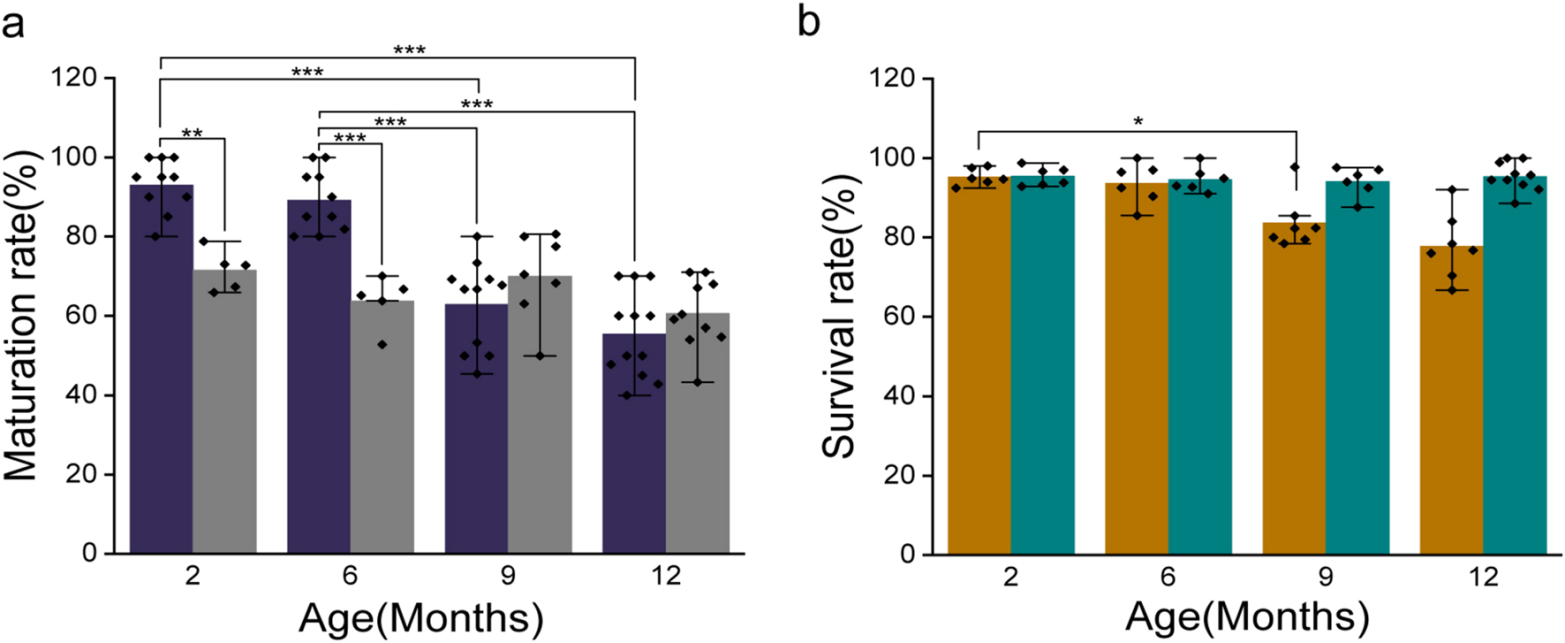
Oocyte maturation potential and vitrification-warming survival. **a** Maturation rate comparison between ovulated (violet) and IVM (grey) oocytes in relation to maternal age. (2O-NV: *N* = 987, 2I-NV: *N* = 1267, 6O-NV: *N* = 598, 6I-NV: *N* = 1006, 9O-NV: *N* = 644, 9I-NV: *N* = 1126, 12O-NV: *N* = 288, 12I-NV: *N* = 1215). **b** Survival rate of vitrified ovulated (orange) and IVM (teal) oocytes 3 h post warming. (2O-V: *N* = 510, 2I-V: *N* = 497, 6O-V: *N* = 320, 6I-V: *N* = 403, 9O-V: *N* = 447, 9I-V: *N* = 434, 12O-V: *N* = 380, 12I-V: *N* = 454). Data represented in Mean ± SEM. *p* < 0.001***, *p* < 0.01**, *p* < 0.05*

The in vivo oocyte maturation rates in 2 and 6 month old females were identical and declined sharply with age (9 and 12 months, *p* < 0.001). However, the IVM potential of oocytes was not influenced by age (Fig. 1a). A similar trend was observed in the percentage of oocytes that survived vitrification-warming. Increasing maternal age resulted in a significant (*p* < 0.05) reduction in the survival rate of the ovulated oocytes. Nevertheless, the survival rate of IVM oocytes remained unchanged in all the age groups (Fig. 1b).

### The Mitochondrial Potential (JC-1 Ratio) of Vitrified Warmed Oocytes was Affected by the Maternal Age

**Fig. 2:**
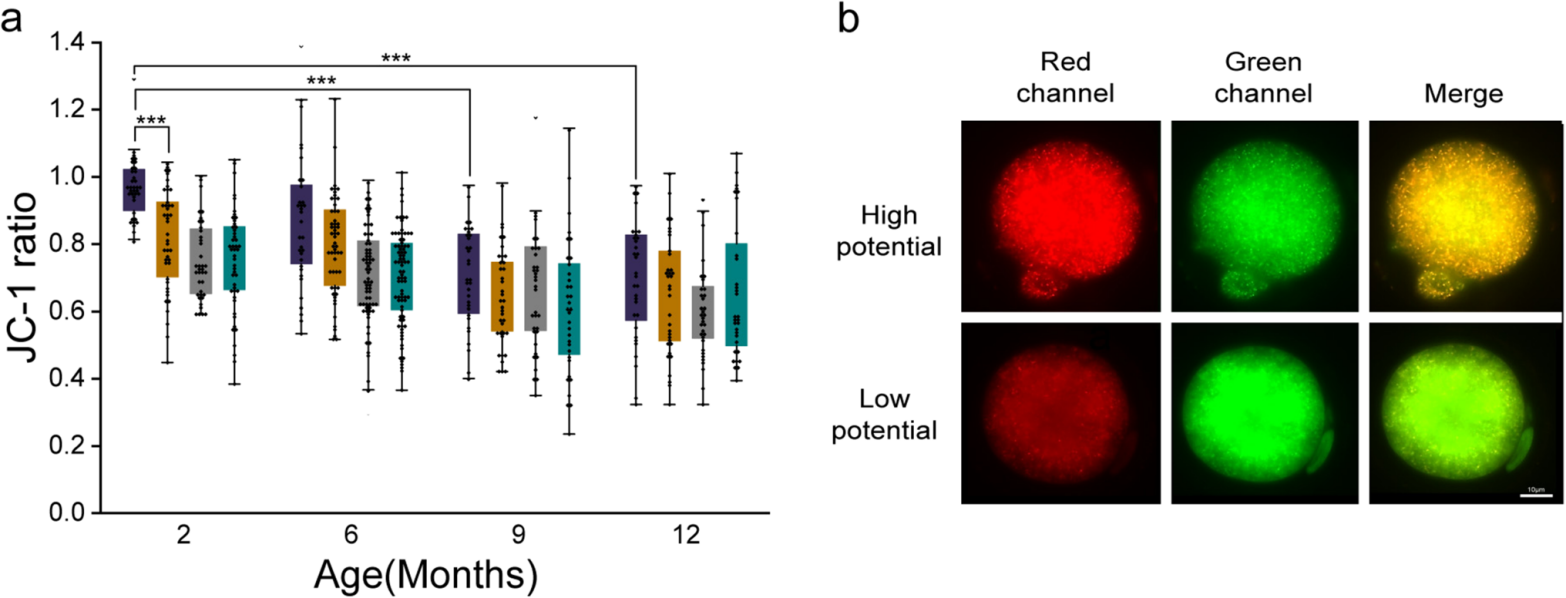
Mitochondrial membrane potential in ovulated and IVM oocytes. **a** Comparison of the mitochondrial potential of O-NV (violet), O-V (orange), I-NV (grey), and I-V (teal) oocytes with maternal age. (2O-NV: *N* = 38, 2O-V: *N* = 43, 2I-NV: *N* = 38, 2I-V: *N* = 48, 6O-NV: *N* = 33, 6O-V: *N* = 50, 6I-NV: *N* = 61, 6I-V: *N* = 51, 9O-NV: *N* = 34, 9O-V: *N* = 37, 9I-NV: *N* = 35, 9I-V: *N* = 34, 12O-NV: *N* = 32, 12O-V: *N* = 34, 12I-NV: *N* = 35, 12I-V: *N* = 32) **b** JC-1 stained fluorescent microscopic images (40X) of oocytes in reducing order of mitochondrial potential: scale bar = 10 µm. Data presented in mean ± SEM derived from three experimental trials. *p* < 0.001***

The impact of maternal age on the mitochondrial function of vitrified ovulated and IVM oocytes was evaluated, as it provides energy for fertilisation and subsequent preimplantation development. Upon vitrification, the JC-1 ratio was comparable between the corresponding non vitrified oocytes across the study groups, except for the 2O-V oocytes (*p* < 0.001) (Fig. 2a). However, JC-1 ratio significantly reduced with increasing maternal age in ovulated oocytes (9O-NV and 12O-NV *vs.* 2O-NV; *p* < 0.001).

### Maternal Age Did Not Affect Meiotic Spindle Integrity of Vitrified-Warmed IVM Oocytes

**Fig. 3:**
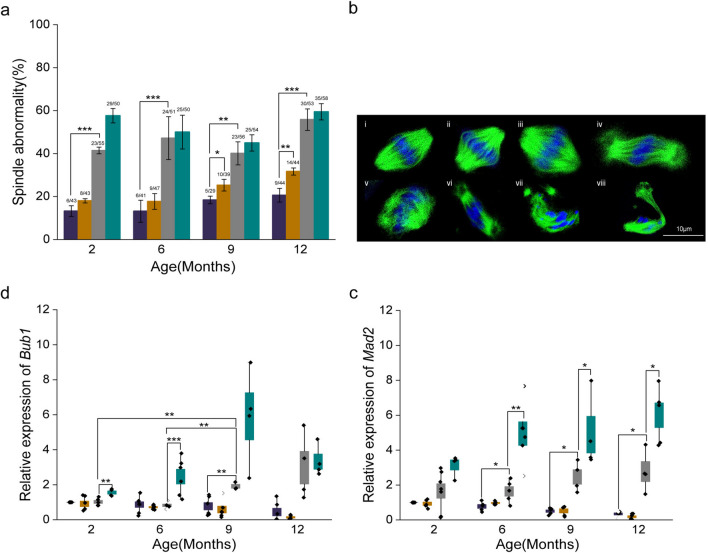
Meiotic spindle structural integrity in ovulated and IVM oocytes. **a** Meiotic spindle abnormalities of O-NV (violet), O-V (orange), I-NV (grey), and I-V (teal) oocytes in relation to maternal age. **b** Confocal microscopic images (100x) of Metaphase-II spindle organisation. The upper panel represents normal (i-iv), and the lower panel (v-vii) represents abnormal spindle organisation. Scale bar = 10 µm. **c**, **d** Relative mRNA expression of spindle check point protein *Bub1* and *Mad2* in O-NV (violet), O-V (orange), I-NV (grey), and I-V (teal) oocytes with maternal age. The expression in the control group was normalised to 1. Data presented in mean ± SEM derived from three experimental trials. *p* < 0.001***, *p* < 0.01**, *p* < 0.05*

Oocyte competence depends on the integrity of the temperature-sensitive spindle organisation. The vitrification-warming process induced significantly higher spindle defects in ovulated oocytes from advanced age (9O-NV (5/29) *vs.* 9O-V (10/39), *p* < 0.05; 12O-NV (9/44) *vs.* 12O-V (14/44), *p* < 0.01). In contrast, the spindle structure of IVM oocytes did not change considerably in any age group after vitrification and warming (Fig. 3a). Meiotic spindle morphology was categorised as normal or abnormal, as depicted in Fig. 3b.

The effect of vitrification-warming on spindle checkpoint proteins was studied by comparing the relative expression levels of *Bub1* (Fig. 3c) and *Mad2* (Fig. 3d) transcripts. An age dependent, non-significant decline in the number of transcripts was observed in ovulated oocytes, which was further reduced in vitrified-warmed oocytes. On the other hand, *Bub1* and *Mad2* transcripts were upregulated in IVM oocytes as maternal age increased, and it further amplified corresponding vitrified-warmed oocytes. *Bub1* demonstrated a statistically significant difference in 2I-V (*p* < 0.01) and 6I-V (*p* < 0.001) compared with the corresponding non vitrified oocytes. The relative expression of *Mad2* was substantially higher in 6I-V (*p* < 0.01), 9I-V (*p* < 0.05), and 12I-V (*p* < 0.05) oocytes than in the corresponding non vitrified IVM oocytes.

### γ-H2AX Foci in Oocytes Were Not Associated With Maternal Age

**Fig. 4:**
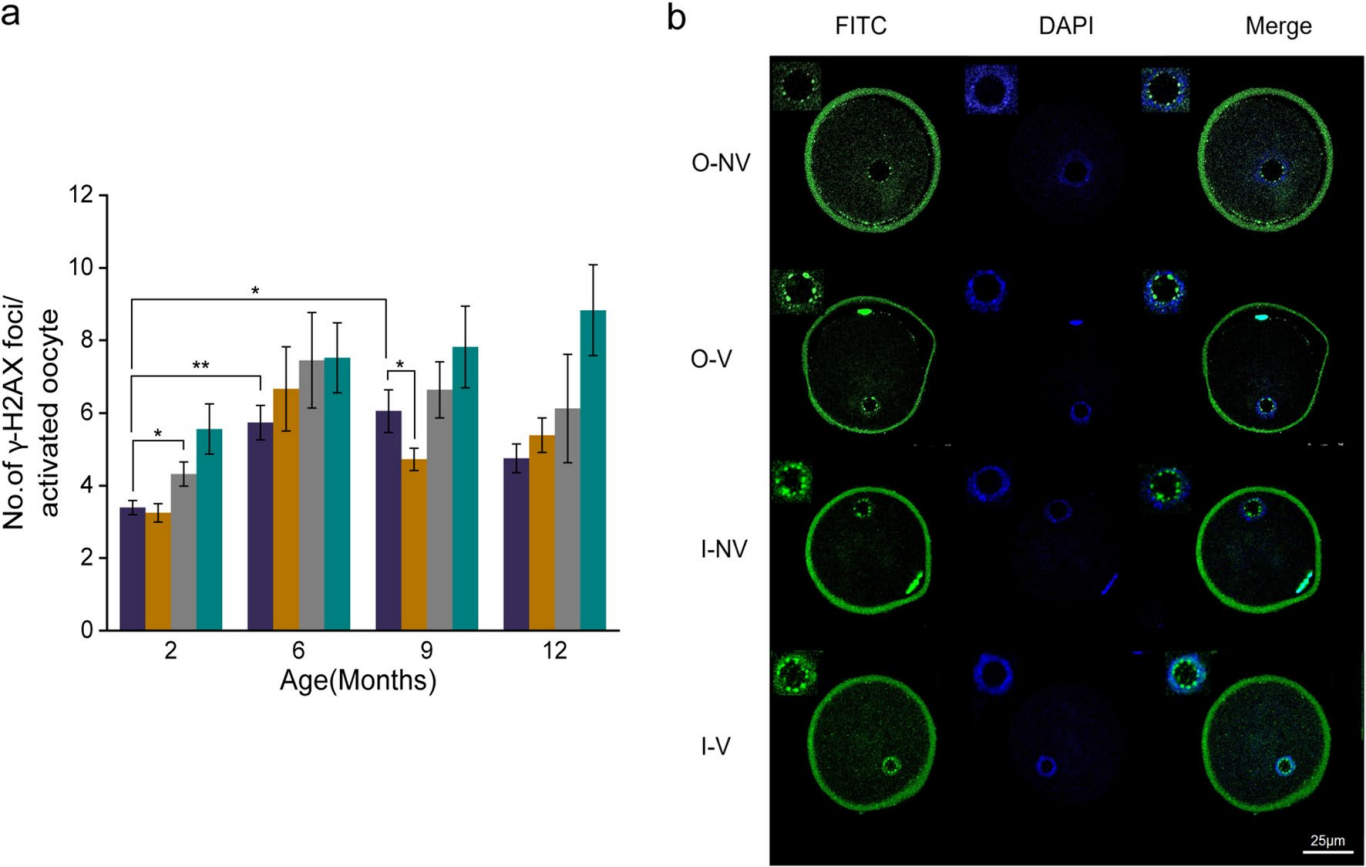
DNA double strand breaks in ovaulated and IVM oocytes. **a** DNA double strand breaks as indicated by **γ-**H2AX foci in O-NV (violet), O-V (orange), I-NV (grey), and I-V (teal) oocytes in relation to maternal age. (2O-NV: *N* = 61, 2O-V: *N* = 44, 2I-NV: *N* = 38, 2I-V: *N* = 34, 6O-NV: *N* = 53, 6O-V: *N* = 42, 6I-NV: *N* = 22, 6I-V: *N* = 25, 9O-NV: *N* = 38, 9O-V: *N* = 36, 9I-NV: *N* = 36, 9I-V: *N* = 26, 12O-NV: *N* = 20, 12O-V: *N* = 18, 12I-NV: *N* = 15, 12I-V: *N* = 16). **b** Confocal microscopic images of **γ-**H2AX foci (100x) in O-NV, O-V, I-NV and I-V activated oocytes. Data presented in Mean ± SEM were derived from three experimental trials. Scalebar = 25 µm. *p* < 0.05*, *p* < 0.01**

The **γ-**H2AX foci are the first indication of DNA double strand breaks. Immunofluorescence analysis of activated oocytes showed that vitrification-warming moderately increased the number of **γ-**H2AX foci in both ovulated and in IVM oocytes. Additionally, ovulated oocytes exhibited age related increase in DNA double strand breaks (2O-NV *vs.* 6O-NV, 9O-NV; *p* < 0.01 and *p* < 0.05 respectively) (Fig. 4a). Figure 4b represents γ-H2AX foci in activated oocytes.

## Discussion

The data presented in this study suggest that advanced maternal age has a negative impact on the cryosusceptibility of ovulated oocytes in mice, as evidenced by the poor survival rate and spindle abnormalities in vitrification-warmed oocytes from advanced age groups. On the other hand, IVM oocyte structure and functional abilities were not significantly impaired by vitrification-warming from different age groups.

The findings of the present investigation were derived by assessing the key functional and structural parameters of oocytes. Mitochondrial membrane potential (ΔΨm) was measured using JC-1, a cationic probe that emits red and green fluorescence based on ΔΨm and is independent of mitochondrial number and size [26]. Meiotic spindle morphology was visualised using antibodies directed against α-tubulin, the fundamental unit of spindle organisation [23]. Further, the spindle checkpoint proteins (*Bub1, Mad2*) that prevent anaphase inception until all chromosomes are attached to the meiotic spindle were analysed by amplifying cDNA in a polymerase chain reaction, as demonstrated earlier [15]. Finally, the effect of vitrification and age on oocyte DNA integrity was examined using the expression of the phosphorylated Ser-139 residue of the histone variant H2AX (γ-H2AX), an early cellular response to the induction of DNA double-strand breaks [27].

Maternal age has a negative impact on oocyte quality, contributing to fertility decline [2, 28, 29]. In the current study, we observed compromised maturation rate, altered mitochondrial potential (JC-1 ratio), disrupted meiotic spindle structure, and DNA damage (γ-H2AX foci) in ovulated oocytes of advanced age groups, which is in agreement with previous clinical and experimental studies [2, 30]. The age-associated reduction in ovulated oocyte efficacy could be attributed to dysfunctional mitochondria, as they are involved in a plethora of cellular events such as nuclear and cytoplasmic maturation, microtubule dynamics, adenosine triphosphate, and protein production [31, 32]. Additionally, the expression of *Bub1* and *Mad2* transcripts downregulated with increasing age in ovulated oocytes, as described in a previous study [15].

Oocyte vitrification is an efficient strategy for elective fertility preservation in age related fertility decline. Even so, the deteriorating oocyte quality in older age groups raises concerns over the oocyte's potential to withstand cryopreservation induced damage. In contrast, a sibling oocytes study in 30–39 year old ICSI patients reported a high survival rate, comparable fertilisation, and embryo development potential of vitrified oocytes. Similarly, a comparable clinical outcome of fresh and vitrified oocyte cycles was found until the age of 39, but not after 40 years [33]. However, a mouse study contradicted human data by demonstrating a lower survival rate and delayed embryo development in oocytes of advanced maternal age [6]. Age-dependent spindle defects and defective mitochondrial dynamics in ovulated oocytes observed in our study may be related to mitochondrial aggregation induced apoptosis in vitrified-warmed advanced age oocytes, and abnormal spindle dynamics have been reported previously [6, 32].

We observed a non-significant reduction in the IVM potential of advanced-age oocytes. The number of oocytes arrested at metaphase I increased with maternal age. The maturation potential was significantly lower than the GVBD rate in 12-month age group oocytes (*p* < 0.05), indicating that genetically compromised oocytes were eliminated during maturation in vitro, although this was not tested in our study. During IVM, GV oocytes undergo spontaneous meiotic resumption owing to an extracorporeal drop in cAMP levels [34]. Meiotic arrest at metaphase I during in vitro maturation was positively associated with DNA damage [18]. The meiotic spindle checkpoint machinery detects DNA damage and stops oocyte progression beyond the M phase if DNA lesions are unrepaired. It is possible that only competent GV oocytes progressed to metaphase II stage, resulting in comparable survival rates of the oocytes regardless of age.

Spindle abnormalities in IVM oocytes were considerably higher than those in the corresponding in vivo group, irrespective of maternal age, suggesting that IVM can significantly impact spindle structure. Although the IVM potential until 6 months was substantially lower than that of in vivo maturation, 9 and 12 months had comparable in vitro and in vivo maturation rates. It is important to note that about 25–35% of oocytes failed to undergo IVM in the 2 and 6 month group. Spindle abnormalities were assessed only in successful IVM oocytes; hence, we cannot ignore that oocytes that failed to progress to metaphase II stage must have had higher spindle defects than those successfully matured.

Vitrification-warming did not impair oocyte survival until 6 months of age. In contrast, the survival rate of IVM oocytes in the advanced age group was moderately higher than that of the corresponding ovulated oocytes (Fig. 1b). It is possible that genetically and functionally compromised oocytes were eliminated during the process of in vitro maturation; hence, matured oocytes had better cryo tolerance. The aberrant expression of *Bub1 and Mad2* transcripts in IVM oocytes suggests that the checkpoint response is more active in protecting the spindle from vitrification-warming, hence not affecting spindle integrity and survival in these oocytes. As spindle integrity deteriorates with increasing maternal age [15, 35] the checkpoint response may help oocytes overcome spindle defects.

In conclusion, our data demonstrated that ovulated oocytes from advanced maternal age have increased susceptibility to vitrification-induced changes compared to oocytes from younger age groups, as indicated by decreased survival rate and increased percentage of abnormal spindles post warming. On the other hand, comparable survival rates and structural integrity evidence that IVM oocytes have an increased ability to tolerate the vitrification-warming process. However, further research is required to understand the phenomenon underlying this interesting and clinically important observation. Significantly, this study is limited in that the findings may not be directly translated to human oocytes. Further investigation is necessary to determine whether human oocytes behave similarly.

### Supplementary Information

Below is the link to the electronic supplementary material.

Supplementary Table 1 List of primers used in SYBR Green qPCR. Actin beta* (Actb), *Glyceraldehyde phosphate dehydrogenase (*Gapdh), *Budding uninhibited by benzimidazole 1(*Bub1), *Mitotic arrest deficient 2 (*Mad2)* (DOCX 17 KB)

## Data Availability

All relevant data are available from the corresponding author upon reasonable request.
