# Supplementary material for: Advanced Maternal Age Affects the Cryosusceptibility of Ovulated but not In Vitro Matured Mouse Oocytes
Source: Reprod Sci. 2024 Jan 31;31(5):1420–8. doi: 10.1007/s43032-024-01462-6 (PMC11090971; doi:10.1007/s43032-024-01462-6)
Supplement: Supplementary file 1 — Supplementary Table 1 List of primers used in SYBR Green qPCR. Actin beta (Actb), Glyceraldehyde phosphate dehydrogenase (Gapdh), Budding uninhibited by benzimidazole 1(Bub1), Mitotic arrest deficient 2 (Mad2) (DOCX 17 KB) [file 43032_2024_1462_MOESM1_ESM.docx]

**Title**

**Advanced maternal age affects the cryosusceptibility of ovulated but not *in vitro* matured mouse oocytes**

**Journal name**

Reproductive sciences

**Authors**

Akshatha Daddangadi^1^, Shubhashree Uppangala^2^, Shama Prasada Kabekkodu^3^ Nadeem Khan G^3^, Guruprasad Kalthur^4^, Riccardo Talevi^5^, Satish Kumar Adiga^1^

**Author affiliations**

^1^Centre of Excellence in Clinical Embryology, Department of Reproductive Science, Kasturba Medical College, Manipal, Manipal Academy of Higher Education, Manipal-576 104, India.

^2^Division of Reproductive Genetics, Department of Reproductive Science, Kasturba Medical College, Manipal, Manipal Academy of Higher Education, Manipal-576 104, India.

^3^Department of Cell and Molecular Biology, Manipal School of Life Sciences, Manipal, Manipal Academy of Higher Education, Manipal-576 104, India

^4^Division of Reproductive Biology, Department of Reproductive Science, Kasturba Medical College, Manipal, Manipal Academy of Higher Education, Manipal-576 104, India.

^5^Dipartimento di Biologia, Università di Napoli "Federico II", Complesso Universitario di Monte S Angelo, Napoli, Italy.

**Corresponding author**

**Satish Kumar Adiga** Centre of Excellence in Clinical Embryology, Department of Reproductive Science, Kasturba Medical College, Manipal, Manipal Academy of Higher Education, Manipal-576 104, E-mail: [satish.adiga@manipal.edu](mailto:satish.adiga@manipal.edu)

| Target mRNA | Strand | Primer Sequence (5’→3’)​ |
| --- | --- | --- |
| *Actb​* | Forward  Reverse | CATTGCTGACAGGATGCAGAAGG  TGCTGGAAGGTGGACAGTGAGG |
| *Gapdh​* | Forward  Reverse | CATCACTGCCACCCAGAAGACTG ATGCCAGTGAGCTTCCCGTTCAG |
| *Bub1* | Forward  Reverse | TGCCACAGTGTGGACCAGAAAC  GACAGTTGGTGATGGCTGCACT​ |
| *Mad2* | Forward  Reverse | CAGAAACTGGTGGTGGTCATCTC  CATCCTGTATGGCTTTCTGGGAC |
